# Supplementary material for: Healthcare Professionals’ Perceptions of and Attitudes towards a Standardized Content Description of Interdisciplinary Rehabilitation Programs for Patients with Chronic Pain—A Qualitative Study
Source: Int J Environ Res Public Health. 2023 Apr 27;20(9):5661. doi: 10.3390/ijerph20095661 (PMC10178188; doi:10.3390/ijerph20095661)
Supplement: Supplementary file 1 [file ijerph-20-05661-s001.zip › ijerph-2284496-supplementary.pdf]

International Journal of
Environmental Research and Public Health

MDPI

Supplementary Materials

Supplement S1: Interview guide

See below the description of the complete interview guide. The responses to the questions in **bold** are included in the analysis for the current paper.

Information to the informant at the start of the interview:

The content description for an IPR program is intended to be completed on one occasion and should provide a general description of the MMR program (that is sustainable over time) and which is suitable for several patients. The clinic can have one and the same IPR program for all the patients or there could be different IPR programs for different patient groups.

Questions regarding informants' background characteristics:

Age: ……………
Sex: …………….

1. **How long have you worked in this healthcare profession?**
2. **How long have you worked with interdisciplinary pain rehabilitation (IPR)?**

Questions about the specific IPR program:

3. Does your clinic already have a content description of your IPR program? Please elaborate…..
4. To what group of patients (diagnosis groups) does the program apply?
5. Do you know the length of the IPR program? In terms of weeks/hours?
6. Do you know how much time (hours) each team member works in the IPR program?
7. Do you have several different IPR programs for different patient groups? Please describe…
8. How could one best describe such a program? In terms of time? Or number of sessions? Or in another way?

Questions about the content in IPR programs:

9. **You have read the template for content descriptions of IPR programs. What do you think about the different parts and questions included?**
10. **What do you think about the relevance of the questions included, taking into consideration your own IPR program as well?**
11. **What do you think about how the questions are phrased? Are they comprehensible/incomprehensible or do they need to be complemented?**
12. **Do you think the content description could describe the IPR program for all patients?**
13. **Is the content description better suited for certain patients? Can you describe which patients?**
14. **Do you think it will be difficult to describe IPR programs considering that programs are supposed to be individually tailored?**
15. Pros and cons with individually tailored IPR programs?
16. **Which parts in a content description do you think are most important for the purpose of describing IPR programs for patients with chronic pain? Elaborate …**

Questions about the informants' attitudes:

17. **What do you think about/ what is your attitude towards using a content description for IPR programs for patients with chronic pain?**
18. **Which positive consequences/advantages could a content description of IPR programs lead to…?**

- **For you?**
- **For the clinic?**
- **For your patients?**

19. **What obstacles/negative consequences could a content description for IPR programs lead to…?**

- **For you?**
- **For the clinic?**
- **For your patients?**

20. **What attitudes do you think your colleagues would have towards a content description for IPR programs for patients with chronic pain?**
21. **What worth or value do you perceive description/standardization/documentation has in the care of patients with chronic pain…?**

- **For you?**
- **For the clinic?**
- **For your patients?**

Supplementary Materials

Supplement S2: Consolidated criteria for reporting qualitative studies (COREQ): 32-item checklist

**No. Item**	**Guide questions/description**	**Reported on Page #**
**Domain 1: Research team and reflexivity**		
Personal Characteristics		
1. Interviewer/facilitator	Which author/s conducted the interview or focus group?	Page 5
2. Credentials	What were the researcher's credentials? E.g. PhD, MD	See Notes on contributors
3. Occupation	What was their occupation at the time of the study?	See Notes on contributors
4. Gender	Was the researcher male or female?	See Notes on contributors (names of authors, 4 women and 3 men)
5. Experience and training	What experience or training did the researcher have?	Page 13 and Notes on contributors
Relationship with participants		
6. Relationship established	Was a relationship established prior to study commencement?	Page 4-5
7. Participant knowledge of the interviewer	What did the participants know about the researcher? e.g. personal goals, reasons for doing the research	Page 4-5, Supplement interview guide
8. Interviewer characteristics	What characteristics were reported about the interviewer/facilitator? e.g. Bias, assumptions, reasons and interests in the research topic	See Notes on contributors, page 4- 5, 13.
**Domain 2: study design**		
Theoretical framework		
9. Methodological orientation and Theory	What methodological orientation was stated to underpin the study? e.g. grounded theory, discourse analysis, ethnography, phenomenology, content analysis	Page 3-5
Participant selection		
10. Sampling	How were participants selected? e.g. purposive, convenience, consecutive, snowball	Page 4
11. Method of approach	How were participants approached? e.g. face-to-face, telephone, mail, email	Page 4-5
12. Sample size	How many participants were in the study?	Page 4
13. Non-participation	How many people refused to participate or dropped out? Reasons?	Page 4
Setting		

14. Setting of data collection	Where was the data collected? e.g. home, clinic, workplace	Page 4
15. Presence of non-participants	Was anyone else present besides the participants and researchers?	No.
16. Description of sample	What are the important characteristics of the sample? e.g. demographic data, date	Page 4
Data collection		
17. Interview guide	Were questions, prompts, guides provided by the authors? Was it pilot tested?	Page 5, Supplement interview guide
18. Repeat interviews	Were repeat interviews carried out? If yes, how many?	No.
19. Audio/visual recording	Did the research use audio or visual recording to collect the data?	Page 5
20. Field notes	Were field notes made during and/or after the interview or focus group?	Page 5
21. Duration	What was the duration of the interviews or focus group?	Page 5
22. Data saturation	Was data saturation discussed?	Page 13
23. Transcripts returned	Were transcripts returned to participants for comment and/or correction?	No.
**Domain 3: analysis and findings**		
Data analysis		
24. Number of data coders	How many data coders coded the data?	Page 5
25. Description of the coding tree	Did authors provide a description of the coding tree?	Page 5, 6<br>Table 2
26. Derivation of themes	Were themes identified in advance or derived from the data?	Page 5
27. Software	What software, if applicable, was used to manage the data?	Page 5
28. Participant checking	Did participants provide feedback on the findings?	No.
Reporting		
29. Quotations presented	Were participant quotations presented to illustrate the themes/findings? Was each quotation identified? e.g. participant number	Page 7 - 10
30. Data and findings consistent	Was there consistency between the data presented and the findings?	Page 7 - 10
31. Clarity of major themes	Were major themes clearly presented in the findings?	Page 7 - 10
32. Clarity of minor themes	Is there a description of diverse cases or discussion of minor themes?	Page 7 - 10
